# Burn out among Iranian dental students: psychometric properties of burnout clinical subtype questionnaire (BCSQ-12-SS) and its correlates

**DOI:** 10.1186/s12909-019-1808-3

**Published:** 2019-10-22

**Authors:** Simin Z. Mohebbi, Reza Yazdani, Musa Talebi, Afsaneh Pakdaman, Marc W. Heft, Hoda Bahramian

**Affiliations:** 1 Research Center for Caries Prevention, Dentistry Research Institute, Tehran University of Medical Sciences, Tehran, Iran; 2 Department of Community Oral Health, School of Dentistry, Tehran University of Medical Sciences, Tehran, Iran; 3 Department of Oral and Maxillofacial Surgery, College of Dentistry, University of Florida, Gainesville, USA

**Keywords:** Burnout, Dental students, Psychometrics, BCSQ-12-SS

## Abstract

**Background:**

Burnout Clinical Subtype Questionnaire (BCSQ-12-SS) is a short valid questionnaire for assessment of burnout in students. The aim of the present study was to evaluate the psychometric properties of Persian-translated version of the BCSQ-12-SS and assess the burn out clinical subtypes and their correlates in dental students.

**Methods:**

In this psychometry study, the BCSQ-12-SS questionnaire in domains of overload (4 questions), lack of development (4 questions), and neglect (4 questions) was translated to Persian and back-translated. Six experts determined the content and face validity of the Persian version. The questionnaire was then piloted on 167 dental students of Tehran University of Medical Sciences in 2016. Data were analyzed using Exploratory Factor Analysis (EFA) and Confirmatory Factor Analysis (CFA) for construct validity and Linear Regression modeling in IBM SPSS and AMOS SPSS. To assess reliability, the questionnaire was filled out by 15 students twice and Kappa coefficient and Composite Reliability (CR) were calculated.

**Results:**

Content validity Ratio (CVR) and Content Validity Index (CVI) values and Cronbach’s alphas were all over 0.8. Kappa coefficient ranged from 65 to 82.5%. The average burnout score was 29.6 out of maximum score of 60. There were no significant differences in burnout scores across the different semesters (8,10 and 12). Financial support by the family significantly affected the total score of burnout and lack of development. In addition, gender, mother’s education, residential status of student, preparing for post graduate exam and financial support by the family affected the overload.

**Conclusion:**

The BCSQ-12-SS has good psychometric properties and therefore can be used to assess burnout in IRANIAN dental students. The BCSQ-12-SS may provide an opportunity to identify individuals at risk for burn out and provide counseling to assist in dental student development.

## Background

Job burnout can be conceptualized as a three-dimensional syndrome comprising emotional exhaustion, depersonalization and reduced personal accomplishment [1]. Emotional exhaustion refers to feelings of emptiness as a component of personal and emotional stress. Depersonalization refers to negative and pessimistic responses or lack of interest in communication with coworkers. Reduced personal accomplishment refers to self-reports of decreased competence and efficiency [2].

Moreover, Maslach et al. (2001) considered job burnout as decreased coping mechanisms to stressors and physical and emotional exhaustion, resulting in negative self-perception, negative attitude towards occupation and lack of communication with others. These symptoms may manifest in a variety of psychological and physical conditions [1].

In studies of university students, burnout has been operationalized as educational burnout which is viewed as exhaustion due to high educational demands and requirements, with individuals having a negative attitude, exhibiting no interest in educational tasks and low efficiency. It is characterized by a feeling of incompetence and mental exhaustion due to not being able to do all the required tasks [3].

Since burnout is often associated with sadness and plays a significant role in decreased interest, learning and function of individuals, it would be important and useful to identify this in students. Furthermore, screening of students, especially dental students, for early signs of burnout may provide opportunities for interventional strategies to prevent the adverse effects on physical and mental health of students.

Physicians and dentists are occupations that are “at risk” for high stress [4]. About 13% of dentists experience high levels of occupational burnout [5, 6].

The 12-question burnout clinical subtype questionnaire (BCSQ-12-SS) was introduced by Montero-Marín et al., in 2011 to measure educational burnout among students of clinical fields including dental students [7, 8]. The three-factor BCSQ-12-SS assesses overload, lack of development and neglect. Overload refers to personal feelings about health risks and personal life experienced by the student and hope for a better future and has a significant association with “Exhaustion”. Lack of development refers to absence of personal development experience for individuals and their interest in other occupations which might allow opportunities to develop their skills. This domain has a significant association with “Cynicism”. Neglect refers to not being responsive to problems and demonstrated inefficiency. This BCSQ-12-SS questionnaire has been validated in Spanish [7, 8]. Before the development of the BCSQ, the most commonly used questionnaire for burnout measusurement was the Maslach Burnout Inventory (MBI) which was longer and was developed for broader application for the assessments of professionals in human services occupations. The BCSQ-12-SS was developed and specifically validated for dental students [7].

## Objectives

This study assessed the psychometric properties of BCSQ-12-SS and their correlates in the Persian translated version administered to Iranian dental students.

## Methods

### Translation and backtranslation, face and content validity

Initially, the BCSQ-12-SS was translated to Persian (See Additional file 1) and was then back-translated to English. The resulting translated English version was then compared with the original English version (See Additional file 2). This step was preceded by taking a written consent from the developer of the questionnaire by email. The final translated version was slightly modified in response to comments from a native English speaker. The resultant version was distributed to six senior faculty of Tehran University of Medical Sciences (five Community oral health experts and one Epidemiologist) to determine its content validity. The mean age of the expert panel was 40 years with at least 5 years of academic teaching experience. Each of these individuals initially evaluated the questionnaire for wording, grammatical points and allocation of items in constructs. To quantitatively assess the content validity, the Content Validity Ratio (CVR) and Content Validity Index (CVI) were calculated. To determine CVR, the experts were requested to rate each item using a three-point scale of “necessary”, “beneficial but not necessary” and “not necessary”.

For the CVI, the same experts were asked to evaluate the remained items based on a 4-point Likert scale on a) simplicity, b) relevancy, and c) clarity. The CVI was calculated by summing up the positive scores for each item divided by the total number of experts. The CVI value of 0.7 or above was considered as threshold value for keeping the items.

Face validity of the questionnaire was also assessed to ensure correct and clear writing and transparency. We applied qualitative method for performing face validity. We asked 10 students to assess each item for vagueness, and difficulty when replying to the questionnaire.

### The cross sectional study and data collection for construct validity

#### Participants and data collection

Backgrounds were inquired by 12 questions on age, gender, marital status (Single, Married), educational semester (8, 10, 12), GPA (Grade Point Average), city of residence (Tehran, Other cities), self- reported financial status (Very Low, Low, Moderate, High, Very high), mothers and father’s level of education (Under diploma, Diploma, Academic education), residential status (Living with parents, In a dormitory, Alone in private home, Shared home with friends), preparing for post graduate exam (Yes, No) and financial support by the family (Inadequate, Good, Very good). The sample size for the pilot cross sectional study to measure the construct validity of the questionnaire was decided according to the Rule of Thumb (Rule of ten) [9], minimum 10 students per variable in factor analysis and by using multiple regression in PASS 11 with an alpha equal to 0.05, Beta of 0.2, R^2^ equal to 0.15, the minimum sample size was estimated as 110–120 for 12 questions and was increased to 209 to cover all willing students in the last 3 years of dental school. These years (4th to 6th year) are allocated to clinical works and all Iranian students were included.

After ensuring optimal content validity (acceptable values over 0.7) and confirming face validity and applying necessary modifications, the questionnaire was completed by 167 dental students (response rate of 80%) of Tehran University of Medical Sciences during the middle of the Semester when they were not involved with a heavy assessment or academic commitment in 2016 to calculate the construct validity of the questionnaire by Exploratory Factor Analysis (EFA), Confirmatory Factor Analysis (CFA), convergent and divergent validity in a census way. An instruction form and a consent form were designed. Information sessions were held for students, and they were briefed about the objectives of the questionnaire. The anonymous questionnaire was then distributed to dental students in one of their didactic classes by one of the researchers, and they were asked to immediately return it. The students rated their level of agreement with each statement in the Persian BCSQ-12-SS questionnaire using a five-point Likert scale from “completely disagree” (1) to “completely agree” (5). The original questionnaire responses had seven-point Likert scale, but according to the expert panel comments, we have changed the scale to five-point Likert scale to localize the questionnaire for Iranian people.

### Statistical analysis

To assess construct validity, Exploratory Factor Analysis (EFA), Confirmatory Factor Analysis (CFA) were performed by means of IBM SPSS statistics version-24 and Amos SPSS version-24 (IBM Corporation, Chicago, USA) software, respectively. Fit indices including ratio of Chi-square to degrees of freedom (X^2^/DF), Root Mean Square Error of Approximation (RMSEA), Comparative Fit Index (CFI), and Non-Normed Fit Index (NNFI) were used. The values of at least 0.90 for CFI, NNFI and below 0.05 for RMSEA indicated good fit (< 0.08 acceptable).

The mean and standard deviation of questionnaire scores were calculated in total and separately for each domain for students in different educational semesters. IBM SPSS statistics version-24 served for descriptive, one way ANOVA, linear regression analyses (Backward method),and calculation of Cronbach’s Alpha and Kappa coefficients. The *p*-value< 0.05 was considered significant.

Standardized estimates equal to or higher than 0.5, Average Variance Extracted (AVE) of 0.5 or higher, and Composite Reliability (CR) of 0.7 or higher were considered to assess convergent validity.

For measuring divergent validity, the AVE for two constructs should exceed their Maximum Shared Variance (MSV).

## Results

One-hundred-sixty-seven dental students (60,5% females, mean age: 23.5 ± 1.20 yrs) completed the BCSQ-12 (Table 1). The response rate was 80%.

**Table 1:** Socio-demographic characteristics of dental students (*n* = 167)

Variables	Mean (SD)	*n*	%
Age	23.45 (1.20)		
Semester	8	36	21.4
	10	62	36.9
	12	69	41.1
Gender	Male	66	39.5
	Female	101	60.5
Father’s education	Under diploma	12	7.2
	diploma	26	15.7
	Academic education	128	77.1
Mother’s education	Under diploma	20	12
	diploma	39	23.4
	Academic education	108	64.7
Income	Very Low	2	1.2
	Low	2	1.2
	Moderate	54	32.1
	High	91	54.8
	Very high	18	10.7
Marital status	Single	119	70.4
	Married	47	28.4
Residential status	With Parents	65	38.9
	Dormitory	67	40.1
	Alone in private home	33	19.8
	Shared home with friends	2	1.2
Financial support by family	Inadequate	13	7.9
	Good	84	50.9
	Very good	68	41.2

The CVI and CVR for each of the 12 questions were calculated and are provided in Table 2.

**Table 2:** CVI and CVR for each question of BCSQ-12-SS

Question	CVI	CVR
1. I think I invest more than is healthy in my commitment to my studies	0.85	0.80
2. I neglect my personal life due to pursuing great objectives in studying	0.80	0.90
3. I am endangering my health in pursuing good results in my studies	0.85	0.95
4. I ignore my own needs to satisfy the requirements of my studies	0.90	0.90
5. I would like to study something else that would be more challenging to my abilities	0.95	0.85
6. I feel that my current studies are hampering the development of my abilities	0.85	0.85
7. I would like to study something else in which I could better develop my talent	0.85	0.80
8. My studies do not provide me with opportunities to develop my abilities	0.85	0.80
9. When the results of my studies are not good at all, I stop making an effort	0.80	0.85
10. I give up in response to an obstacle in my studies	0.90	0.90
11. I give up when faced with any difficulty in my tasks as a student	0.85	0.85
12. When the effort invested in studying is not enough, I give up	0.80	0.90

All the CVR and CVI values were quite good (between 0.80 and 0.95), and the 12 questions were entered in the construct validity phase. Some minor changes were performed in qualitative approach to receive the experts comments on content and face validity of the questions and consequently two questions underwent minor changes in grammar.

### Psychometric properties of the questionnaire in Persian

After Exploratory Factor Analysis, the final rotated matrix of the components Dimensions is shown in Table 3.

**Table 3:** Final Rotated Component Matrix for BCSQ-12-SS in dental students

Questions	Component		
	1	2	3
q1	−.13	.06	.70^c^
q2	.10	.14	.84^c^
q3	.09	< −.01	.82^c^
q4	.13	.06	.79^c^
q5	.05	.86^b^	.06
q6	.13	.85^b^	.15
q7	.13	.92^b^	−.02
q8	.15	.90^b^	.08
q9	.82^a^	.09	.13
q10	.90^a^	.10	.04
q11	.92^a^	.15	< .01
q12	.87^a^	.11	.02

^a^Components of Factor 1 ^b^Components of Factor 2 ^c^Components of factor 3

The educational form of BCSQ has been designed in three domains of overload (4 questions:q1-q4), lack of development (q5-q8) and neglect (q9-q12) with 12 questions. The goodness-of-fit indices of first order CFA for each of three domains of burn out were as following:

Overload: (CFI = 0.96; NNFI = 0.80; X2/DF = 8.56 (*P* < 0.001); RMSEA = 0.21), Lack of developmemt: (CFI = 0.99; NNFI = 0.96; X2/DF = 3.86 (*P* = 0.02); RMSEA = 0.13), and Neglect: (CFI = 0.99; NNFI = 0.98; X2/DF = 2.41(*P* = 0.09); RMSEA = 0.09).

**Fig. 1:**
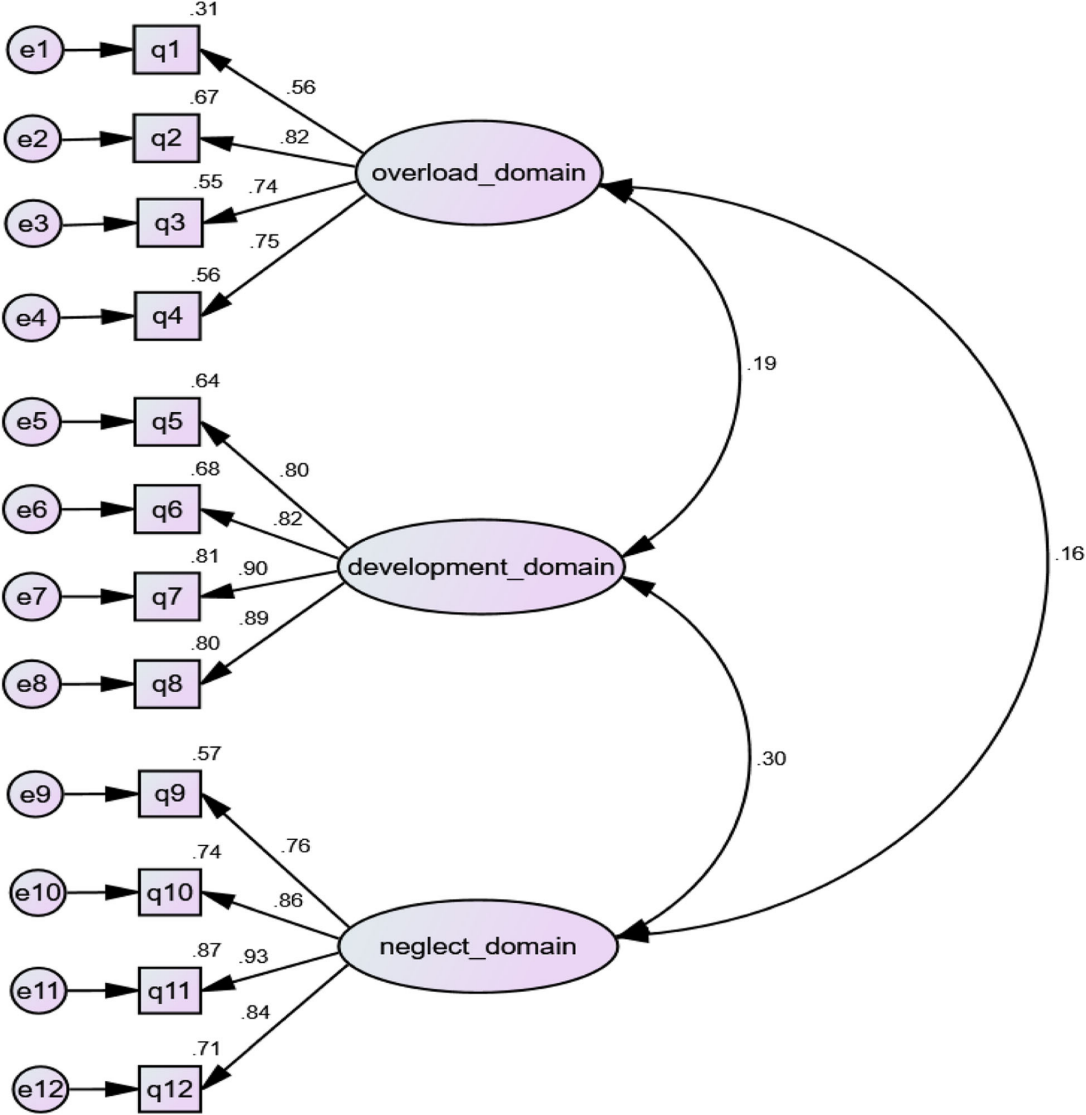
Final pattern of 12-Item BCSQ Questionnaire Following CFA with Standardized Coefficients Illustrated on the Arrows

For the second order CFA in final model, the following initial values were obtained:

CFI = 0.97, NNFI = 0.96, X2/DF = 1.75 (*P* < 0.001), RMSEA = 0.07. The final model of confirmatory factor analysis along with the standard coefficients is shown in Fig. 1.

All the factor loadings for latent constructs were significant (*P* < 0.001) and more than 0.5 (Fig. 1) and The AVEs of all constructs were more than 0.5 and CR (Composite Reliability) was more than 0.7, suggesting convergent validity (Table 4). The AVEs of all constructs were greater than their MSVs, suggesting divergent validity (Table 4). The reproducibility of the three-factors was quite good (Cronbach’s alpha was 0.80 for overload (questions 1 to 4), 0.91 for lack of development (questions 5 to 8) and 0.91 for the neglect domain (questions 9 to 12) and for the whole questionnaire, it was 0.85).

**Table 4:** Convergent and Divergent Validity and Reliability Indices of the Burn-out questionnaire Constructs on 167 students

	AVE	MSV	CR	Cronbach Alpha
overload_domain	0.52	0.03	0.81	0.80
Lack of development_domain	0.73	0.09	0.91	0.91
neglect_domain	0.72	0.09	0.91	0.91

Test-retest reproducibility was assessed for 15 students (Kappa coefficient ranged from 65 to 82.5% and CR were between 0.81 to 0.91 (Table 4)).

### Dental students burn out score, subtypes and their associating factors

Out of a maximum score of 60, the mean (SD) educational burnout score of dental students in semester 8, 10 and 12 was 32.17 (8.35), 28.85 (7.98),and 28.93 (6.63), respectively. The average total burnout score was 29.6. There were no significant differences in total burnout scores across the different semesters (*F* = 2.70, *P* = 0.07).

Table 5 shows the mean and standard deviation of the scores for dental students in different semesters for each of the three domains.

**Table 5:** Mean (SD) scores acquired by dental students (*n* = 167) in different semesters in the three domains of burn out

Domain	Semester						
	8		10		12		*P*-value
	Mean	SD	Mean	SD	Mean	SD	
Overload	11.30	3.36	12.26	3.79	11.78	3.36	0.257
Lack of development	11.58	4.94	8.89	4.27	9.45	4.22	0.031
Neglect	9.56	4.03	7.71	2.81	7.69	2.41	0.006

The 75th percentile for total burn out score was 34 and for each domain of BCSQ-12-SS was 15, 13 and 9 respectively. (Table 6).

**Table 6:** Descriptive statistics for the total Burn out score and sub-scales of Overload, Lack of development, and Neglect

	Mean	SD	Median	Minimum	Maximum	Percentile 75	interquartile range	Skewness	kurtosis
Overload	11.8	3.6	29	4	20	15	5.5	< 0.01	−0.53
Lack of development	9.7	4.5	11	4	20	13	7	0.52	−0.55
Neglect	8.1	3.1	9	4	20	9	3	1.02	2.19
Total burn out score	29.6	7.6	8	12	60	34	9	0.58	1.70

Based on the results of regression analysis controlling for backgrounds (age, gender, marital status, educational semester, GPA, city of residence, self- reported financial status, mothers and father’s level of education, residential status, preparing for post graduate exam and financial support by the family), only the effects of financial support by the family on the burnout score remained significant and, in particular, the lower the financial support, the higher the burnout score (Table 7, P: 0.033). Other backgrounds including the GPA of students were not associated with total burn out score (*P* > 0.05).

**Table 7:** Regression test to assess the effect of variables on ^a.^burn out score, ^b.^overload score, and ^c.^lack of development score

Variables	Non-standard coefficients		Standard coefficient	*P*-value
	B	Standard error	Beta	
^a.^ Burn out score				
Financial support by the family	−2.51	1.12	−0.21	0.033
^b.^ Overload Score				
Gender	1.52	0.73	0.20	0.039
Mother’s level of education	−0.96	0.48	− 0.27	0.047
Residential status	0.79	0.40	0.18	0.050
preparing for post graduate exam	2.34	0.75	0.29	0.002
Financial support by the family	−1.23	0.57	−0.21	0.033
^c.^ lack of development score				
Financial support by the family	−1.24	0.64	−0.18	0.050

Gender, the residential status of student and preparing for post graduate exam was associated with higher overload scores. However, mother’s level of education, and financial support by the family was associated with lower overload scores (*P* < 0.05). (Table 7).

With regards to the development domain, only financial support by the family had a significant effect on score of this domain (Table 7, *P* < 0.05). None of the variables had any significant effect on the score of neglect domain (*P* > 0.05).

## Discussion

Occupational (and educational) burnout has gained increasing interest in the recent years. This study was the first to assess psychometric properties of Persian version of BCSQ-12-SS questionnaire in dental students. The results demonstrate that it is suitable tool for assessment of educational burnout among Persian dental students. Several widely used goodness-of-fit indices demonstrated that the Confirmatory factor model with three domains fit the data. This questionnaire has Construct, Convergent and Divergent validity and is reliable to apply for Iranian dental students.

With input from an expert panel, we changed the response scale of the original questionnaire with seven-point Likert scale to five-point Likert scale to better adapt the questionnaire for Iranian people as in Persian language, no important data could be obtained by one to seven in comparison to one to five Likert scale. It likely provides minimal impact on the response variability.

The majority of respondents were young females. They were mostly from families with good socioeconomic status. The responses of the students to questions of the BCSQ-12-SS were highly variable. The Cronbach’s alpha and Kappa’s reliability showed acceptable internal consistency and reproducibility of students’ responses to 12 questions. Thus, this tool may provide an expedient screen for dental students as regard to burnout and enable therapeutic interventions based on personal characteristics of individuals.

Montero-Marin et al. in 2012 evaluated educational burnout based on individual differences using BCSQ-12-SS and concluded that due to simplicity and optimal efficacy, it is suitable for screening and identifying those who might benefit from primary counseling [10]. The participants above the 75th percentile (P75) of the BCSQ-12-SS score, are considered as “high scores”, whereas those with scores below the 75th percentile are considered to have “low scores” [7]. According to our result, all of the students were involved with kind of burn out which might limit their capability to learn.

The prevalence of educational burnout among dental students in Iran has been rarely evaluated and reports from other countries have shown mixed results. Mafla et al. (2015) evaluated educational burnout and its correlation with different factors using the Maslach Burnout Inventory-Student Survey (MBI-SS) among dental students in Colombia and showed that only 7% of students had the educational burnout criteria [11]. Campos et al. (2012) evaluated the prevalence of educational burnout syndrome among dental students in Brazil using Maslach’s educational burnout questionnaire and showed that 17% of students had educational burnout [12]. Moreover, Alemany-Martinez et al., in 2008 evaluated educational burnout among post-graduate dental students using the Maslach Burnout Inventory in Barcelona University (oral surgery and implant, orthodontics and comprehensive dentistry) and showed that high levels of educational burnout were present in 2–3% of the students [13].

Difference in prevalence of educational burnout may be related to the questionnaires used. Most previous studies used Maslach’s questionnaire for this purpose. Some researchers used the primary form of educational burnout questionnaire with two subscales of emotional exhaustion and cynicism. In some other studies, general questionnaires, which are not specific for students, were used [14].

Based on the results of the current study, the GPA of students were not associated with their burn out score. However, some studies showed that students with high GPA had lower educational burnout score [15]. Students with educational burnout are desperate, irritable and hopeless and are not interested in studying and have lost motivation. Such students less commonly participate in class activities and show poorer educational performance. The correlation of educational burnout with educational performance is bi-directional. In other words, higher GPA score decreases burnout and higher burnout can decrease performance and GPA score of students [16]. Lee et al. (2010) showed that students with high GPA score have higher self-esteem and show greater resistance against problems. They less commonly show burnout [17]. Nikodijevic et al. (2012) concluded that 54.4% of students with low GPA were at risk of occupational burnout while 26.6% were at high risk of burnout [18].

Based on the results of regression test of our study, educational semester had no significant effect on burnout score (neither in total score nor in the score of the three domains). It is in contrast to some studies that showed students in higher semesters experience greater educational burnout due to a more intense curriculum and stresses related to clinical work on patients. For instance, Dyrbye et al. in 2006 found out that concerns regarding the future, fear of doing something wrong, having inadequate skills and high expectations of parents result in higher stress and greater exhaustion in senior compared to junior dental students [19].

Despite all the above, students in higher semesters have more experience in their training and know how to overcome their stresses. This might help them cope and decrease their educational burnout, which may be the probable reason of different results of our study.

Galan et al. (2014) evaluated the educational burnout in the 2nd, 4th and 5th year dental students and reported that the prevalence of educational burnout was higher among the 2nd and 4th year dental students compared to the 5th year students [20]. Kogoj et al. (2014) used Oldenburg burnout inventory questionnaire among medical and dental students in Ljubljana medical university in Slovenia and reported that students in higher semesters had higher burnout scores [21].

In our study, the students’ residential status had a significant effect on the overload score of questionnaire. It appears that living alone (versus living with parents) is associated with higher level of educational burnout because students living alone may no longer have the support of family and thus, due to increased roles and higher responsibilities, they are subject to educational burnout.

Age of dental students had no significant effect on overload score in our study. The reason may be the fact that students participated in our study were all in a close age range. The same result was obtained in previous studies [22, 23]. Singh et al. (2016) evaluated the main factors related to occupational burnout among dentists and dental students in a systematic review and reported the common factors related to occupational burnout to be age, male gender, student’s residential status, long working hours, high workload, involvement in clinical scoring system and personality types [24]. Previous studies have reported the same factors related to occupational burnout [8, 25, 26].

Montero-Marin et al. (2011) reported that gender had no significant effect on the results of the university employees [8]. Galan et al. (2014) found no significant difference in educational burnout of male and female dental students [20]. Rajpurohit et al. (2015) evaluated burnout syndrome in dental students of two universities using MBI-SS questionnaire and found no significant difference between males and females [27]. Their finding was in line with ours which indicates that stresses of girls competing with boys no longer exist and females now have an equal performance with males in fields in which, males used to be dominant [24].

Based on the results of regression, the socioeconomic status of the family and level of education of parents had no significant effect on BCSQ-12-SS questionnaire. However, the effect of financial support by the family on the total score of questionnaire and overload domain was significant. This indicates the significance of financial support in prevention of burnout. It is further speculated that level of income is an indicator of optimal overall quality of life (in financial aspect). By an increase in quality of life, mental health indices also improve. Also, by a decrease in financial aspect of quality of life (determined by level of income), health status may deteriorate.

Based on the results of regression test, marital status had no significant effect on educational burnout of students. However, it seems that married students have less problems than single students, which may be due to family support provided for married students. On the other hand, married students have higher responsibilities, which may interfere with their educational progression. Mafla et al. (2015) evaluated Colombian dental students and reported that students in higher semesters, older students and married students had higher level of educational burnout [11]. Torabi Parizi et al. (2014) found no significant association between occupational burnout and demographic factors such as age, gender, work experience (years), marital status, weekly working hours and private or public work among dentists applying MSI questionnaire [28]. Despite its popularity, the validity of the Maslach Burnout Inventory (MBI) – in all its versions – has been the subject of considerable debate. This conceptualization of burnout, operationalized through the “Burnout Clinical Subtype Questionnaire”, is very useful for the specific evaluation of the syndrome and for the design of treatment strategies depending on the characteristics of each clinical case. Given that it provides a broader framework that exceeds the possibilities for evaluation and intervention implicit rather than the standard design of the MBI, which is more directed towards a unified (although three-dimensional) definition of the syndrome [9, 28, 29].

The strength point of our study was to introduce a newly developed short educational burnout questionnaire with proper psychometric properties in Persian. Our study had a psychometric cross-sectional design and data were collected in a short period of time; thus, a cause and effect relationship could not be determined in our study. Due to the above-mentioned factor and the fact that our study was conducted in a single university, we may not generalize our results to the entire dental students in Iran. Therefore, we suggest more comprehensive studies to measure students burnout in different dental schools and provision of more comparative results to prepare the context for interventional studies.

## Conclusion

The BCSQ-12-SS has optimal psychometric properties and therefore can be used to determine the level of educational burnout in dental students. By use of this questionnaire, students can be screened for burnout and necessary interventions may be designed and implemented for them. The existence of burn out among dental students regardless of study year calls for more efforts to preserve mental health by councelling and programming for relief of the study barriers.

## Supplementary information

**Additional file 1.** Persian BCSQ-12-SS Questionnaire. Translated Persian version

**Additional file 2.** English BCSQ-12-SS Questionnaire. Original English version

## Data Availability

The datasets used and/or analyzed during the current study are available from the corresponding author on reasonable request.

## Abbreviations

AVE: Average Variance Extracted
BCSQ-12-SS: Burnout Clinical Subtype Questionnaire
CFA: Confirmatory Factor Analysis
CFI: Comparative Fit Index
CR: Composite Reliability
CVI: Content Validity Index
CVR: Content Validity Ratio
EFA: Exploratory Factor Analysis
GPA: Grade Point Average
MSI: Maslach Burnout Inventory
MSV: Maximum Shared Variance
NNFI: Non-Normed Fit Index
RMSEA: Root Mean Square Error of Approximation
X2/DF: Ratio of Chi-square to Degrees of Freedom
