# Supplementary material for: Burn out among Iranian dental students: psychometric properties of burnout clinical subtype questionnaire (BCSQ-12-SS) and its correlates
Source: BMC Med Educ. 2019 Oct 22;19:388. doi: 10.1186/s12909-019-1808-3 (PMC6805565; doi:10.1186/s12909-019-1808-3)
Supplement: Supplementary file 1 — Additional file 1. Persian BCSQ-12-SS Questionnaire. Translated Persian version [file 12909_2019_1808_MOESM1_ESM.docx]

- **File name : Additional file 1**
- **File format : DOCX**
- **Title of data: Persian BCSQ-12-SS Questionnaire ‎**
- **Description of data: Translated Persian version**

**Additional file 1:**

**Persian BCSQ-12-SS Questionnaire**

پرسشنامه ترجمه شده به فارسی **BCSQ-12-SS**

دانشگاه علوم پزشکی و خدمات بهداشتی درمانی تهران

دانشکده‌ی دندانپزشکی

عنوان طرح

روان‌سنجی پرسشنامه زیرگروه‌های بالینی فرسودگی تحصیلی BCSQ-12-SS در دانشجویان دانشکده دندانپزشکی دانشگاه تهران

دانشجوی گرامی، پرسش‌نامه‌ای که پیش رو دارید برای *سنجش فرسودگی تحصیلی و زیرشاخه‌های آن* در دانشجویان دندانپزشکی است. این پرسشنامه بدون نام بوده وپر کردن آن اختیاری است. تنها برای سنجش میزان و نوع فرسودگی تحصیلی می‌باشد و اطلاعات شما محرمانه میماند. بدیهی است همکاری شما، در این طرح ما را در جهت نیازسنجی و تامین نیازهای حرفه‌ای دانشجویان و بهبود شرایط روحی ایشان حین تحصیل یاری می‌کند. لذا خواهشمند است نسبت به تکمیل دقیق‌تر پرسشنامه و برگرداندن آن همکاری فرمایید.

**سوالات زمینه‌ای**

1. سن: . . . . . . . . . . . سال

1. جنس: مرد ⃝ زن ⃝
2. وضعیت تاهل: مجرد ⃝ متاهل ⃝
3. در حال حاضر در ترم چندم مشغول به تحصیل هستید؟ . . . . . . . . . . . .
4. آخرین معدل: . . . . . . . . .
5. شهر محل زندگی خانواده: . . . . . . . . . . .
6. وضعیت اقتصادی خانواده: بسیار خوب ⃝ خوب ⃝ متوسط ⃝ ضعیف ⃝ بسیار ضعیف ⃝
7. میزان تحصيلات پدر: بیسواد ⃝ ابتدایی/راهنمایی ⃝ دبیرستان و دیپلم ⃝ فوق دیپلم/ لیسانس ⃝ فوق لیسانس و بالاتر ⃝
8. میزان تحصيلات مادر: بیسواد ⃝ ابتدایی/راهنمایی ⃝ دبیرستان و دیپلم ⃝ فوق دیپلم/ لیسانس ⃝ فوق لیسانس و بالاتر ⃝

10.در حال حاضر کجا سکونت دارید؟ با والدین ⃝ خوابگاه ⃝ خانه مستقل ⃝ خانه مشترک با دوستان ⃝

11. آیا در حال درس خواندن جهت شرکت در امتحان دستیاری هستید؟ بله ⃝ خیر ⃝

12. حمایت مالی از طرف خانواده چگونه است؟ ناکافی ⃝ خوب ⃝ خیلی خوب ⃝

1. چه نوع دانشجویی هستید؟ آزاد ⃝ سهمیه ⃝ تکمیلی ⃝

**پرسشنامه‌ی BCSQ-12-SS**

| ردیف | عبارات | کاملاً مخالف | مخالف | مطمئن نیستمم | موافق | کاملاً موافق |
| --- | --- | --- | --- | --- | --- | --- |
| 1 | من فکر می‌کنم سلامتم را در جهت انجام وظایف تحصیلی‌ام صرف می‌کنم. |  |  |  |  |  |
| 2 | من به دلیل دنبال کردن اهداف بزرگ در تحصیل از زندگی شخصیم غفلت می‌کنم. |  |  |  |  |  |
| 3 | من برای رسیدن به نتایج خوب در تحصیل، حتی سلامتی‌ام را به خطر می‌اندازم. |  |  |  |  |  |
| 4 | من برای برآورده کردن الزامات تحصیلی‌ام نیازهای خودم را نادیده می‌گیرم. |  |  |  |  |  |
| 5 | من میل دارم در رشته دیگری که توانایی‌های مرا بیشتر به چالش بکشاند تحصیل کنم |  |  |  |  |  |
| 6 | من احساس می‌کنم رشته تحصیلی فعلیم، مانع توسعه توانایی‌هایم می‌باشد |  |  |  |  |  |
| 7 | من ترجیح می‌دهم در رشته دیگری تحصیل کنم تا استعدادهایم را بهتر شکوفا کند. |  |  |  |  |  |
| 8 | رشته تحصیلی من فرصت شکوفایی توانمندی‌های مرا فراهم نمی‌کند. |  |  |  |  |  |
| 9 | زمانی که نتایج تحصیلم اصلاً خوب نیستند، دست از هر گونه تلاشی می‌کشم. |  |  |  |  |  |
| 10 | من در مواجهه با موانع در تحصیلم دست از ادامه کار برمی‌دارم. |  |  |  |  |  |
| 11 | من در صورتی که در انجام وظایفم به عنوان یک دانشجو با هرگونه مشکلی روبرو شوم دست از ادامه کار برمی‌دارم. |  |  |  |  |  |
| 12 | زمانی که تلاشی که در تحصیل صرف کرده‌ام کافی نیست، از ادامه کار دست برمی‌دارم. |  |  |  |  |  |

با تشکر
