# Supplementary material for: Burn out among Iranian dental students: psychometric properties of burnout clinical subtype questionnaire (BCSQ-12-SS) and its correlates
Source: BMC Med Educ. 2019 Oct 22;19:388. doi: 10.1186/s12909-019-1808-3 (PMC6805565; doi:10.1186/s12909-019-1808-3)
Supplement: Supplementary file 2 — Additional file 2. English BCSQ-12-SS Questionnaire. Original English version [file 12909_2019_1808_MOESM2_ESM.docx]

**‎ File name : Additional file 2**

**• File format : DOC‎X**

**• Title of data: English BCSQ-12-SS Questionnaire ‎**

**• Description of data: Original English version ‎**

**Additional file 2:**

**English BCSQ-12-SS Questionnaire**

**Overload**

1. I think I invest more than is healthy in my commitment to my studies

2. I neglect my personal life due to pursuing great objectives in studying

3. I am endangering my health in pursuing good results in my studies

4. I ignore my own needs to satisfy the requirements of my studies

**Lack of development**

5. I would like to study something else that would be more challenging to my abilities

6. I feel that my current studies are hampering the development of my abilities

7. I would like to study something else in which I could better develop my talent

8. My studies do not provide me with opportunities to develop my abilities

**Neglect**

9. When the results of my studies are not good at all, I stop making an effort

10. I give up in response to an obstacle in my studies

11. I give up when faced with any difficulty in my tasks as a student

12. When the effort invested in studying is not enough, I give up
